# *Ginkgo biloba* Extract EGb 761 Improves Vestibular Compensation and Modulates Cerebral Vestibular Networks in the Rat

**DOI:** 10.3389/fneur.2019.00147

**Published:** 2019-02-25

**Authors:** Magdalena Lindner, Astrid Gosewisch, Eva Eilles, Christina Branner, Anja Krämer, Rosel Oos, Eckhard Wolf, Sibylle Ziegler, Peter Bartenstein, Thomas Brandt, Marianne Dieterich, Andreas Zwergal

**Affiliations:** ^1^German Center for Vertigo and Balance Disorders, DSGZ, Ludwig-Maximilians-University of Munich, Munich, Germany; ^2^Department of Nuclear Medicine, Ludwig-Maximilians-University of Munich, Munich, Germany; ^3^Department of Veterinarian Medicine, Ludwig-Maximilians-University of Munich, Munich, Germany; ^4^Munich Cluster of Systems Neurology, SyNergy, Munich, Germany; ^5^Clinical Neuroscience, Ludwig-Maximilians-University of Munich, Munich, Germany; ^6^Department of Neurology, Ludwig-Maximilians-University of Munich, Munich, Germany

**Keywords:** unilateral vestibulopathy, vestibular compensation, *Ginkgo biloba* extract EGb 761, small animal PET, vertigo, pharmacotherapy, *in vivo* cerebral imaging

## Abstract

Unilateral inner ear damage is followed by behavioral recovery due to central vestibular compensation. The dose-dependent therapeutic effect of *Ginkgo biloba* extract EGb 761 on vestibular compensation was investigated by behavioral testing and serial cerebral [^18^F]-Fluoro-desoxyglucose ([^18^F]-FDG)-μPET in a rat model of unilateral labyrinthectomy (UL). Five groups of 8 animals each were treated with EGb 761-supplemented food at doses of 75, 37.5 or 18.75 mg/kg body weight 6 weeks prior and 15 days post UL (groups A,B,C), control food prior and EGb 761-supplemented food (75 mg/kg) for 15 days post UL (group D), or control food throughout (group E). Plasma levels of EGb 761 components bilobalide, ginkgolide A and B were analyzed prior and 15 days post UL. Behavioral testing included clinical scoring of nystagmus, postural asymmetry, head roll tilt, body rotation during sensory perturbation and instrumental registration of mobility in an open field before and 1, 2, 3, 5, 7, 15 days after UL. Whole-brain [^18^F]-FDG-μPET was recorded before and 1, 3, 7, 15 days after UL. The EGb 761 group A (75 mg/kg prior/post UL) showed a significant reduction of nystagmus scores (day 3 post UL), of postural asymmetry (1, 3, 7 days post UL), and an increased mobility in the open field (day 7 post UL) as compared to controls (group E). Application of EGb 761 at doses of 37.5 and 18.75 mg/kg prior/post UL (groups B,C) resulted in faster recovery of postural asymmetry, but did not influence mobility relative to controls. Locomotor velocity increased with higher plasma levels of ginkgolide A and B. [^18^F]-FDG-μPET revealed a significant decrease of the regional cerebral glucose metabolism (rCGM) in the vestibular nuclei and cerebellum and an increase in the hippocampal formation with higher plasma levels of ginkgolides and bilobalide 1 and 3 days post UL. Decrease of rCGM in the vestibular nucleus area and increase in the hippocampal formation with higher plasma levels persisted until day 15 post UL. In conclusion, *Ginkgo biloba* extract EGb 761 improves vestibulo-ocular motor, vestibulo-spinal compensation, and mobility after UL. This rat study supports the translational approach to investigate EGb 761 at higher dosages for acceleration of vestibular compensation in acute vestibular loss.

## Introduction

Unilateral damage to the peripheral vestibular organ induces characteristic signs of vestibular imbalance, including spontaneous nystagmus, head tilt, and falling to the lesion side. Even if the damage to the labyrinth persists, initial symptoms recover over days to weeks due to central vestibular compensation (VC). VC is not a unique global process but comprises various structural and functional processes with separate and distinct time courses. It implicates neuronal plasticity in various hubs of cerebral and spinal vestibular networks (1–4). Most important regions for reorganization are the vestibular nuclei, the vestibulocerebellum, the thalamus and hippocampus (3–7). Mechanisms of VC can be examined on a cellular and neuronal level by various histological and electrophysiological techniques or on a whole brain network level using *in vivo* methods like serial positron emission tomography (PET) (8–10). Characteristic changes of regional cerebral glucose metabolism (rCGM) have been depicted recently after unilateral labyrintectomy (UL) in the rat using [^18^F]-Fluoro-desoxyglucose ([^18^F]-FDG)-μPET (9). The asymmetry of rCGM in the vestibular nuclei, vestibulocerebellum, thalamus and temporoparietal cortex directly after UL was followed by an adjustment of rCGM in the vestibular nuclei, rCGM increase in the ipsilesional spinal trigeminal nucleus, and increased vestibulocerebellar rCGM over time (9). [^18^F]-FDG-μPET imaging was also feasible to quantify rCGM changes in cerebral vestibular networks by pharmacological interventions with 4-aminopyridine and N-acetyl-DL-leucine following inner ear damage (11, 12).

A possible therapeutic strategy for acute unilateral vestibular disorders is to improve and accelerate VC by medication such as betahistine, N-acetyl-DL-leucine or *Ginkgo biloba* extract EGb 761 [for review see (13)]. The pharmacon EGb 761 is a standardized extract of *Ginkgo biloba*, which contains flavonoids and terpene lactones at defined doses (14, 15). Previous studies have shown improvement of vestibular symptoms by EGb 761 after unilateral vestibular damage in rats, cats, and guinea pigs (16–18). Placebo-controlled trials in patients with unilateral peripheral and central vestibular lesions gave first evidence for an augmentation of exercise-induced effects on VC by EGb 761 (19). However, the mode of action and the pharmacological substrate by which the polyvalent EGb 761 extract improves VC are still not clear. Experimental data from neurodegenerative animal models suggest multiple effects of EGb 761 on neural plasticity, including modulation of long-term potentiation, neuronal spine morphology and density, neurito- and neurogenesis mostly by terpene lactones (20). The aim of this study was to determine systematically dose-, substrate-, and time-dependent effects of EGb 761 on vestibulo-ocular motor, vestibular-spinal, locomotor, and orientational symptoms after UL in the rat by behavioral testing. To further elucidate the EGb 761 mode of action during VC, serial [^18^F]-FDG-μPET was performed at various stages in the course of compensation. We hypothesized that EGb 761 accelerates compensation of spontaneous nystagmus and postural asymmetry and improves mobility after UL. We expected EGb 761-induced rCGM changes to appear at various central vestibular network levels during VC.

## Methods

### Animals

All animal experiments were approved by the government of Upper Bavaria and performed in accordance with the guidelines for the use of living animals in scientific studies and the German Law for the Protection of Animals. Male Sprague-Dawley rats (mean 400 ± 20 g, age 3 months at time of UL, Charles River Ltd, UK) were housed two animals per cage in a temperature- and humidity-controlled room with a 12 h light/dark cycle, with free access to food and water. In total 46 animals were included in the study.

### EGb 761 Food

Phytoestrogen-free food with and without *Ginkgo biloba* extract EGb 761 supplement was provided by Altromin Spezialfutter GmbH & Co. KG, Germany. EGb 761® was provided by Dr. Willmar Schwabe Pharmaceuticals, Karlsruhe, Germany. It is a dry extract from *Ginkgo biloba* leaves (35–67:1) with extraction solvent: acetone 60% (w). The extract is adjusted to 22–27% Ginkgo flavonoids calculated as Ginkgo flavone glycosides, 5–7% terpene lactones consisting of 2.8–3.4% ginkgolides A, B, C, and 2.6–3.2% bilobalide and contains <5 ppm ginkgolic acids^1^. Food contained either EGb 761 in a dose of 735 or 367.5 mg or 184 mg per kg food or no supplement (control food). Food was prepared in 10 mm palletization and color-coded to avoid carry-over or mix-up (EGb 761 food: yellow, control food: green). Animals were allowed to consume the food *ad libitum*. Food was exchanged weekly and the extent of food consumption documented. Based on mean food uptake per body weight, 735, 367.5, and 184 mg EGb 761 per kg food equated to 75, 37.5, and 18.75 mg per kg body weight and day.

### Experimental Design

**Figure 1:**
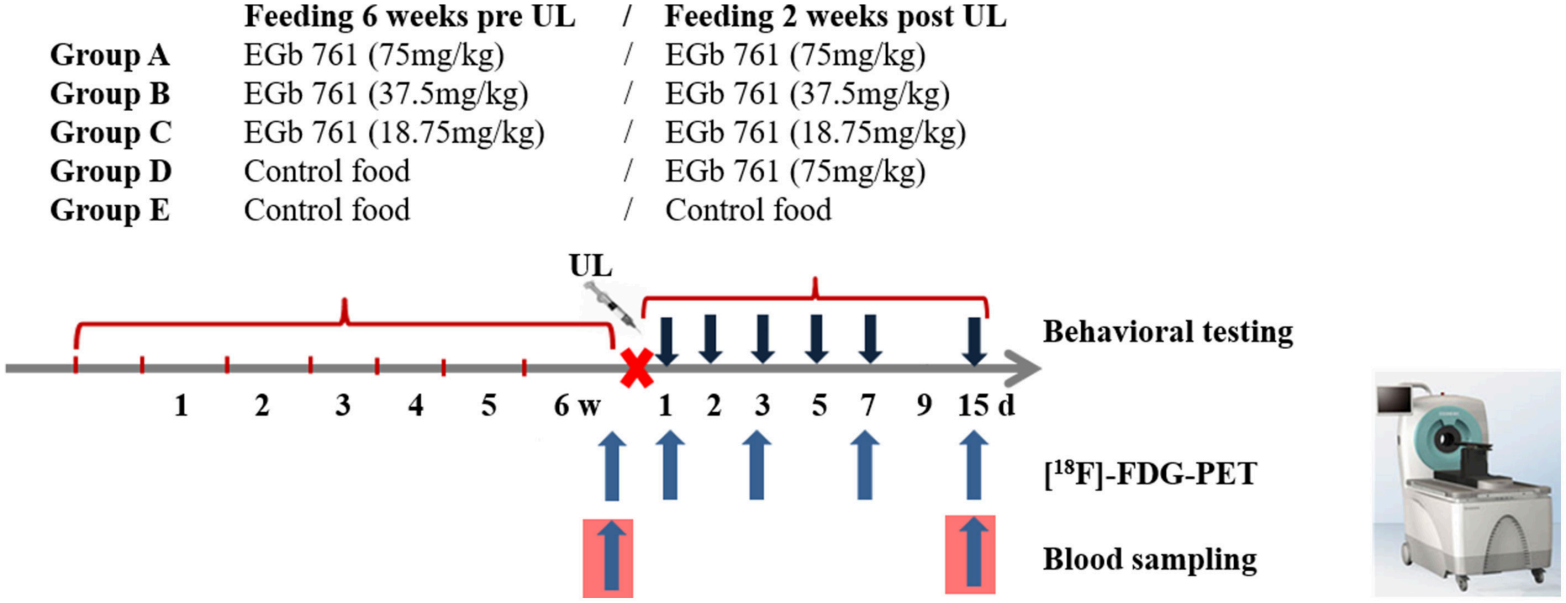
Study design and time course of treatment, behavioral testing, and [^18^F]-FDG-μPET-scanning. Groups of 8 rats were treated with EGb 761 food at a dose of 75 mg/kg (group A), 37.5 mg/kg (group B), or 18.75 mg/kg body weight (group C) 6 weeks prior and 15 days post UL. Another group of 8 rats was treated with control food prior and EGb 761 food (75 mg/kg body weight) for 15 days post UL (group D). Finally, a group received control food throughout (group E). Clinical scoring for vestibular imbalance and automated analysis of locomotor and spatial exploration behavior was performed on days 1, 2, 3, 5, 7, and 15 post UL. Cerebral glucose metabolism was depicted by serial [^18^F]-FDG-μPET at baseline and days 1, 3, 7, and 15 post UL. Blood samples were drawn before UL and 15 days post UL. UL, unilateral labyrinthectomy; [^18^F]-FDG-PET, [^18^F]-Fluorodeoxyglucose-positron-emission tomography; w, weeks; d, days.

Different food application protocols were tested to investigate effects of dose and treatment duration: (1) Eight animals received EGb 761 at a dose of either 75 mg/kg (group A), 37.5 mg/kg (group B), or 18.75 mg/kg body weight (group C) per day 6 weeks prior to and 15 days post UL. (2) Eight animals were treated with EGb 761-free control food 6 weeks prior to and EGb 761 food (75 mg/kg body weight per day) 15 days post UL (group D). (3) Eight animals received control food 6 weeks prior to and 15 days post UL (group E). All animals underwent behavioral testing by clinical scoring of vestibular imbalance and instrumental analysis of locomotion before and on days 1, 2, 3, 5, 7, and 15 after UL. Sequential whole-brain [^18^F]-FDG-μPET was recorded prior to and 1, 3, 7, and 15 days post UL. Blood samples were drawn before and 15 days after UL in all animals and analyzed for the EGb 761 components bilobalide, ginkgolide A and B (Figure 1).

### Chemical Unilateral Labyrinthectomy

Chemical unilateral labyrinthectomy was performed as described earlier (9, 21–23). Animals were anesthetized with 1.5% isoflurane in O_2_ delivered up to 1.2 l/min O_2_ via a mask. For surgical analgesia 1.5 mg/kg meloxicam was injected s.c. before and 3 days after surgery. An additional 5 ml saline was injected s.c. as a bolus. After local anesthesia with 1% bupivacaine hydrochloride, a left paramedian incision was made to expose the lamboidal ridge and the external ear canal. The external ear canal was opened just anterior to the exit point of the facial nerve. With a 26-gauge needle the tympanic membrane was perforated caudally to the hammer shaft, and about 0.150 ml of a 20% bupivacaine solution was instilled into the tympanic cavity. After about 2 min the bupivacaine solution was aspirated and instilled slowly again (three repetitions in total). After the local anesthesia was instilled, the same procedure was followed to instill 0.150 ml of a 10% solution of p-arsanilic acid, which irreversibly desensitized the primary sensory cells of the inner ear (23). After the last thorough aspiration, the wound was closed by skin suture and for preventive antibiosis 2 mg/kg marbofloxacin was injected s.c. for 3 days.

### Criteria for Exclusion

Animals were excluded from the study if the following symptoms were observed:

- loss of body weight equal to more than 20% of the pre-treatment value
- ulcer of the cornea, which could occur due to an inadvertent lesion of the facial nerve during UL
- bleeding from the tympanic cavity, which could prevent the diffusion of bupivacaine or p-arsanilic acid into the inner ear
- abnormalities in behavioral scoring, i.e., convulsions, paresis, hemiataxia, etc.

Based on these criteria, four animals had to be excluded.

### Clinical Scoring of Vestibular Asymmetry After UL

Behavioral symptoms of vestibular imbalance were scored by two experienced veterinarians blinded for the treatment condition for four components after unilateral vestibular ablation [based on (24)]: nystagmus, postural asymmetry, head roll tilt, and elevation tail test. Scores were depicted as mean values of two raters. Each component was given a score of up to 10:

- *Nystagmus* was observed visually. Intensity of spontaneous nystagmus was scored with 6–10 points, with 1 point for every 60 beats per minute (bpm). In the absence of spontaneous nystagmus at rest, the animal was touched slightly. If nystagmus was evoked, it was scored 1–5 points, with 1 point for every 60 bpm.
-*Postural deficits* were scored as follows: spontaneous barrel rolling−10 points; barrel rolling evoked by light touch or air-puff−9 points; recumbent position on lesion side without leg support−8 points; some ipsilesional leg support−7 points; moving around on one side or using ipsilesional legs for recumbent support−6 points; moving around with bilateral leg support−5 points; moving around with occasional falls to the ipsilesional side−4 points; moving around leaning toward the ipsilesional side−3 points; hardly noticeable asymmetry−2 points; postural asymmetry only noticeable when picked up−1 point.
-Spontaneous *head roll tilt* was scored by estimating the angle between the jaw plane and the horizontal, with 10 points given either for a 90° angle or if the animal rested recumbent on the lesion side or showed barrel-rolling toward that side. Seven points correlated to a 60° and 5 points to a 45° angle.
-Influence of perceptive somatosensory input to postural control during VC was examined by the *elevation tail test* (ETT). Animals were picked up from the ground at the radix of their tail and body rotation was scored by estimating in degrees, with 10 points given for more than 360°, 8 points for 180–360°, 6 points for <180° and 0 points for no relevant rotation.

### Instrumental Locomotor Analysis

Testing of locomotor behavior was performed in the open field using automated video tracking (EthoVision System, Noldus, Netherlands). Animals were allowed to move freely in the rectangular open field (side length 70 cm) over 10 min. Position of nose, body center, and tail were automatically detected by video software. Mean locomotor velocity, cumulative duration of movement, changes in mobility state, frequency of rotation, and cumulative duration of stay in the center or at the edges of the maze were quantified. Locomotor velocity was taken as an overall estimate of mobility, the time in center zone as a measure of mobility and spatial exploration behavior. Heat maps of position at place were generated to indicate overall movement in open space.

### Blood Sampling and Substrate Analysis

Blood samples were taken before and 15 days after UL in all animals (500 μl in mean). Blood sampling was restricted to these time points to avoid side effects of a regular haemodilution. The blood was collected in vials that were coated with lithium heparin and centrifuged immediately (10 min/2,000 rotations per min). The plasma was separated and mixed with 1% hydrogen chloride (10% of obtained plasma volume). The samples were stored at −20°C. Analysis for bilobalide and ginkgolide A and B was performed using liquid chromatography-electrospray ionization-tandem mass spectrometry as described earlier (25).

### μPET Imaging

Anesthesia was induced with isoflurane (as described above) and a cannula was placed in a tail vein. Before the animals awakened from anesthesia, a [^18^F]-FDG (50 MBq) bolus was injected (in 0.5 ml saline) and the animals were allowed to move freely until anesthesia was induced again with isoflurane (1.8%) for the μPET-scan. The scan was started 30 min after [^18^F]-FDG injection. Animals were positioned in the Siemens Inveon PET scanner (Siemens Healthineers, Erlangen, Germany) and were kept warm with a heating pad. In order to avoid head movement, the head position was fixed using a custom-made head-holder. A 30-min-long emission recording was initiated followed by a 7 min transmission scan using a rotating [^57^Co] point source. Upon recovery from anesthesia the rats were returned to their home cages.

### Image Processing and Statistical Analysis

Emission recordings were reconstructed with iterative reconstruction employing the Ordered Subsets Expectation Maximization (OSEM-3D) algorithm which includes scatter and attenuation correction (Siemens Healthineers, Erlangen, Germany) and results in a final 128 × 128 × 159 matrix. For attenuation correction, the corresponding transmission measurements at the end of the emission scan were used. The voxel dimensions of the reconstructed images were 0.59 × 0.59 × 0.79 mm^3^. [^18^F]-FDG distribution in the reconstructed images was used as measure of regional cerebral glucose metabolism (rCGM). Data processing and statistical analysis were performed by means of custom-made protocols implemented in the statistical parametric mapping software SPM8 (Wellcome Department of Cognitive Neurology, Great Britain). Using automated algorithms, all individual [^18^F]-FDG-μPET images were stereotactically normalized co-registered to a custom-made [^18^F]-FDG template (average of 20 healthy rats), which was manually co-registered to a digital high-resolution cryosection-based atlas of rat brain. [^18^F]-FDG data were normalized to the mean activity value in a whole-brain atlas region, in order to remove differences in the individual count levels. Images were compared in a voxel-wise manner between time points and conditions using SPM8. A correlation analysis of rCGM with individual plasma levels of ginkgolide A, ginkgolide B and bilobalide across the EGb 761 groups A, B and C was performed on day 1 and 3 (using plasma levels before UL) and day 15 post UL (with plasma levels from the same day).

### Statistics

IBM SPSS Statistics (version 23.0; SPSS, Chicago, IL) was used for all statistical tests. Kolmogorov-Smirnov-Test indicated that none of the parameters (nystagmus, postural asymmetry, head roll tilt, elevated tail test, locomotor velocity, time in center-zone) was normally distributed. Statistical group comparison for behavioral scoring was performed by the non-parametric Kruskal-Wallis H test and *post hoc* testing with Bonferroni correction to analyse significant differences between time points and groups after UL. Blood levels for bilobalide, ginkgolides A and B at baseline and 15 days after UL were compared between time points and groups using the non-parametric Kruskal-Wallis H test and *post hoc* testing with Bonferroni correction. Pearson's coefficient of correlation was calculated between individual values for plasma levels of bilobalide, ginkgolides A and B (mean of baseline and post-UL values) and the slope of locomotor velocity, nystagmus and postural asymmetry over time, respectively. For each rat the average change of locomotor velocity and postural asymmetry per day was computed by the slope of the linear function starting on day 2 and ending at day 15 post UL.

## Results

### High-Dose EGb 761 Improves Vestibular Imbalance, Locomotor and Exploratory Behavior After UL

**Figure 2:**
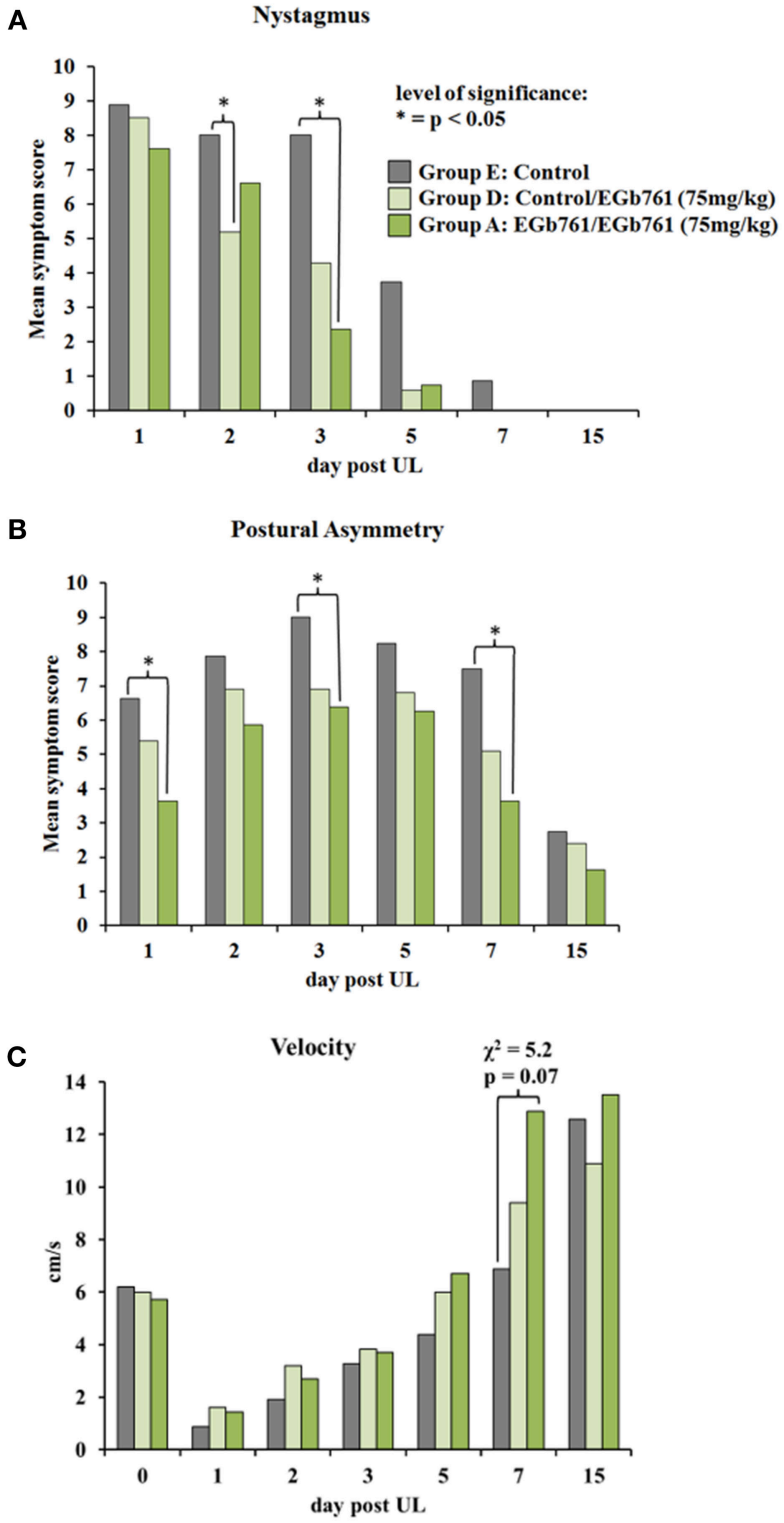
Effects of EGb 761 on clinical scores of vestibular imbalance and locomotor velocity. **(A)** Nystagmus scores were significantly lower in the EGb 761/EGb 761 group (75 mg/kg) (group A) on day 3 and in the Control/EGb 761 group (75 mg/kg) (group D) on day 2 post UL compared to Controls (group E). **(B)** In the EGb 761/EGb 761 75 mg/kg group A postural asymmetry scores were significantly lower on days 1, 3 and 7 post UL relative to the Control group. **(C)** The Kruskal-Wallis H test between groups A, D and E showed only some trend to a different locomotor velocity on day 7 (χ^2^ = 5.2, *p* = 0.07). Solid lines and asterisk indicates a level of significance of *p* < 0.05 in Kruskal-Wallis H *post hoc* test.

After UL, animals showed severe signs of vestibular imbalance including spontaneous nystagmus, postural asymmetry, head roll tilt and body turns on elevated tail rotation test. In all groups, nystagmus peaked at day 1 (Control group: 8.9; Control/EGb 761: 8.5; EGb 761/EGb 761 75 mg/kg: 7.6) and disappeared completely in the first 7 days post UL. However, for group comparison the Kruskal-Wallis H test showed a significant difference of nystagmus scores on day 2 (χ^2^ = 14.2, *p* = 0.007) and day 3 (χ^2^ = 14.2, *p* = 0.007). *Post hoc* tests indicated a decrease of nystagmus in the Control/EGb 761 group D vs. Control group E on day 2 (*z* = 17.1, *p* = 0.023) and the EGb 761/EGb 761 75 mg/kg (group A) relative to the Control group E on day 3 (*z* = 21.4, *p* = 0.003). Nystagmus scores in the EGb 761/EGb 761 (group A) and Control/EGb 761 (group D) were not statistically different on all days (Figure 2A). Postural asymmetry increased in all groups within the first 3 days due to delayed toxicity effects of p-arsanilic acid. In all groups, the postural asymmetry scores decreased significantly from day 3 until day 15 post UL (Control group E: peak mean score on day 3: 9.0 vs. mean score on day 15: 2.8, *p* = 0.003; EGb 761/EGb 761 75 mg/kg (group A): peak mean score on day 3: 6.3 vs. mean score on day 15: 1.6, *p* = 0.002; Control/EGb 761 (group D): peak mean score on day 3: 7.0 vs. mean score on day 15: 2.4, *p* < 0.001). Intergroup comparison showed a significant difference of postural asymmetry scores between groups on day 1 (χ^2^ = 16.9, *p* = 0.002), day 3 (χ^2^ = 15.4, *p* = 0.004), and day 7 post UL (χ^2^ = 12.9, *p* = 0.012). *Post hoc* tests with Bonferroni correction revealed a significant decrease of postural asymmetry scores in the EGb 761/EGb 761 (group A) relative to the Control group E on day 1 (*z* = 19.6, *p* = 0.002), day 3 (*z* = 20.0, *p* = 0.009) and day 7 (*z* = 19.25, *p* = 0.015) and a significant reduction of scores for the Control/EGb 761 group D vs. the Control group E on day 3 post UL (*z* = 15.8, *p* = 0.05) (Figure 2B). The course of postural compensation accelerated by about 7 days in the EGb 761/EGb 761 group A. Head roll tilt persisted until day 15 post UL in all groups. Kruskal-Wallis H test showed no significant difference between groups at any time point, except a tendency on day 3 (χ^2^ = 9.0, *p* = 0.061). Head roll tilt scores on day 3 were lower in the EGb 761/EGb 761 group A (9.1 ± 0.7) and in the Control/EGb 761 group D (9.2 ± 0.6) compared to the Control group E (10.0 ± 0.0). The elevated tail rotation test also persisted to be pathological with mean scores above 7 until day 15 post UL in all groups. Scores in the EGb 761/EGb 761 group A and in the Control/EGb 761 group D were not statistically different from the Control group E in the Kruskal-Wallis H test for the different time points (data not shown).

After UL, mean locomotor velocity and cumulative duration of movement decreased in the Control, EGb761/EGb 761 (75 mg/kg), and Control/EGb761 (75 mg/kg) group (groups E, A, D) as compared to baseline. Over time both parameters increased in all groups significantly and were above baseline level after day 7 post UL. The Kruskal-Wallis H test between groups A, D and E showed only some trend to a different locomotor velocity on day 7 (χ^2^ = 5.2, *p* = 0.07). Mean locomotor velocity in the EGb 761/EGb 761 group A was more than double as high than the Control group E (13.9 ± 2.8 cm/s vs. 6.8 ± 1.5 cm/s) and higher than the Control/EGb 761 group D (9.4 ± 2.2 cm/s) (Figure 2C).

**Figure 3:**
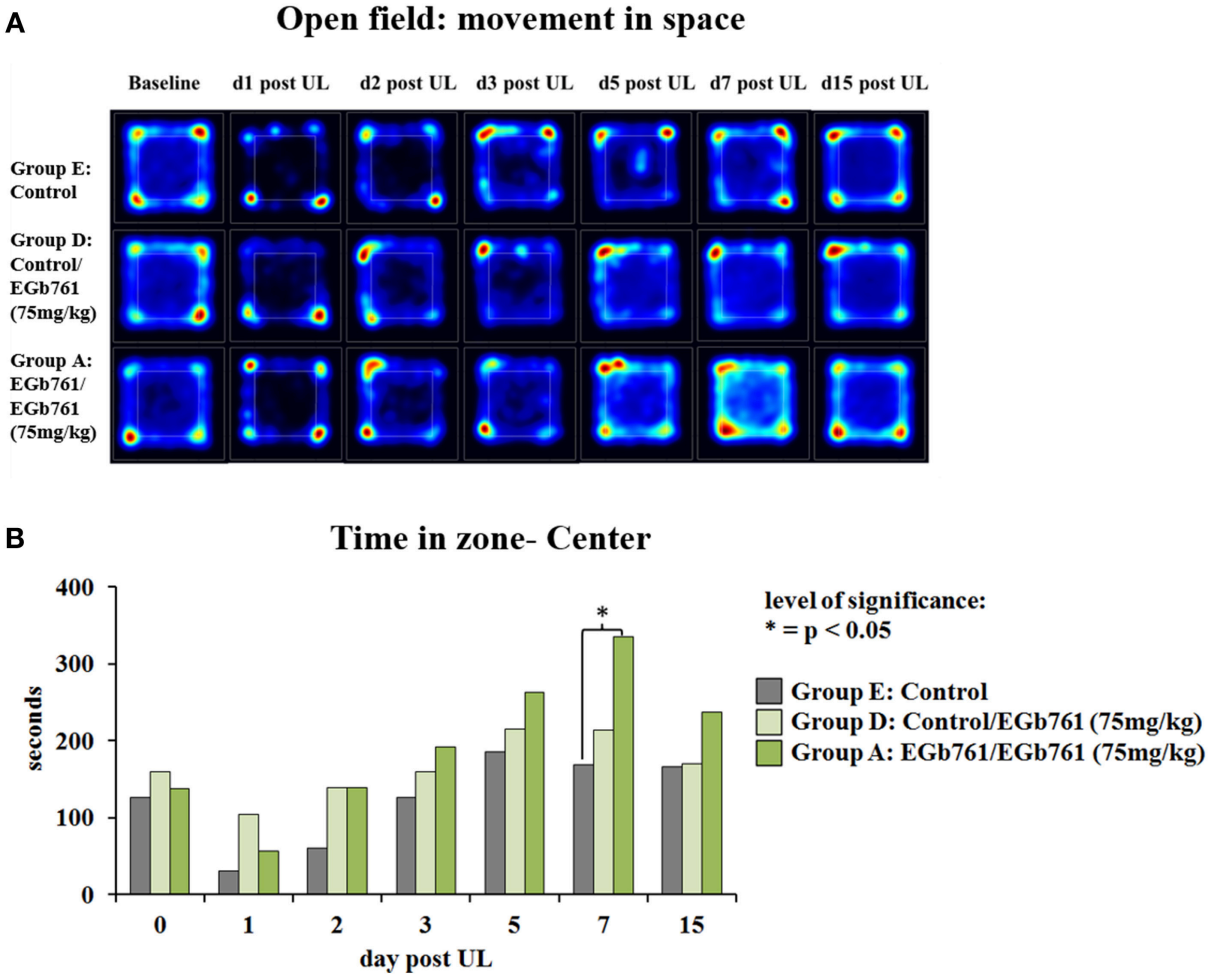
Effects of EGb 761 on spatial exploration behavior in the open field. **(A)** Analysis of spatial exploration patterns in the open field indicated that on days 1, 2, and 3 after UL rats in all groups stayed mostly at the border zone. After day 3 post UL, the EGb 761/EGb 761 75 mg/kg group A showed an increased exploration in the central zone of the open field. Heatmaps indicate the cumulative time at place (blue to red scale increasing duration). **(B)** Cumulative time in the central zone was significantly higher on day 7 post UL in the EGb 761/EGB 761 75 mg/kg group A as compared to Controls group E. Asterisk indicates a level of significance in Kruskal-Wallis *post hoc* test of *p* < 0.05; d, day; UL, unilateral labyrinthectomy.

Analysis of spatial exploration patterns in the open field indicated that on days 1, 2, and 3 after UL rats in all groups stayed mostly at the border zone. After day 3 post UL, the EGb 761/EGb 761 75 mg/kg (group A) showed a statistically increased exploration in the central zone of the open field (Figure 3A). Cumulative time of stay in the central zone was significantly different between groups A, D and E on day 7 post UL (χ^2^ = 16.6, *p* = 0.002). *Post hoc* test revealed a higher duration of in center stay for the EGb 761/EGb 761 75mg/kg (group A) vs. the Control group (*z* = 19.62, *p* = 0.014) (Figure 3B).

### Dose-Dependency of EGb 761 Effects on Vestibular Imbalance and Locomotor Velocity

**Figure 4:**
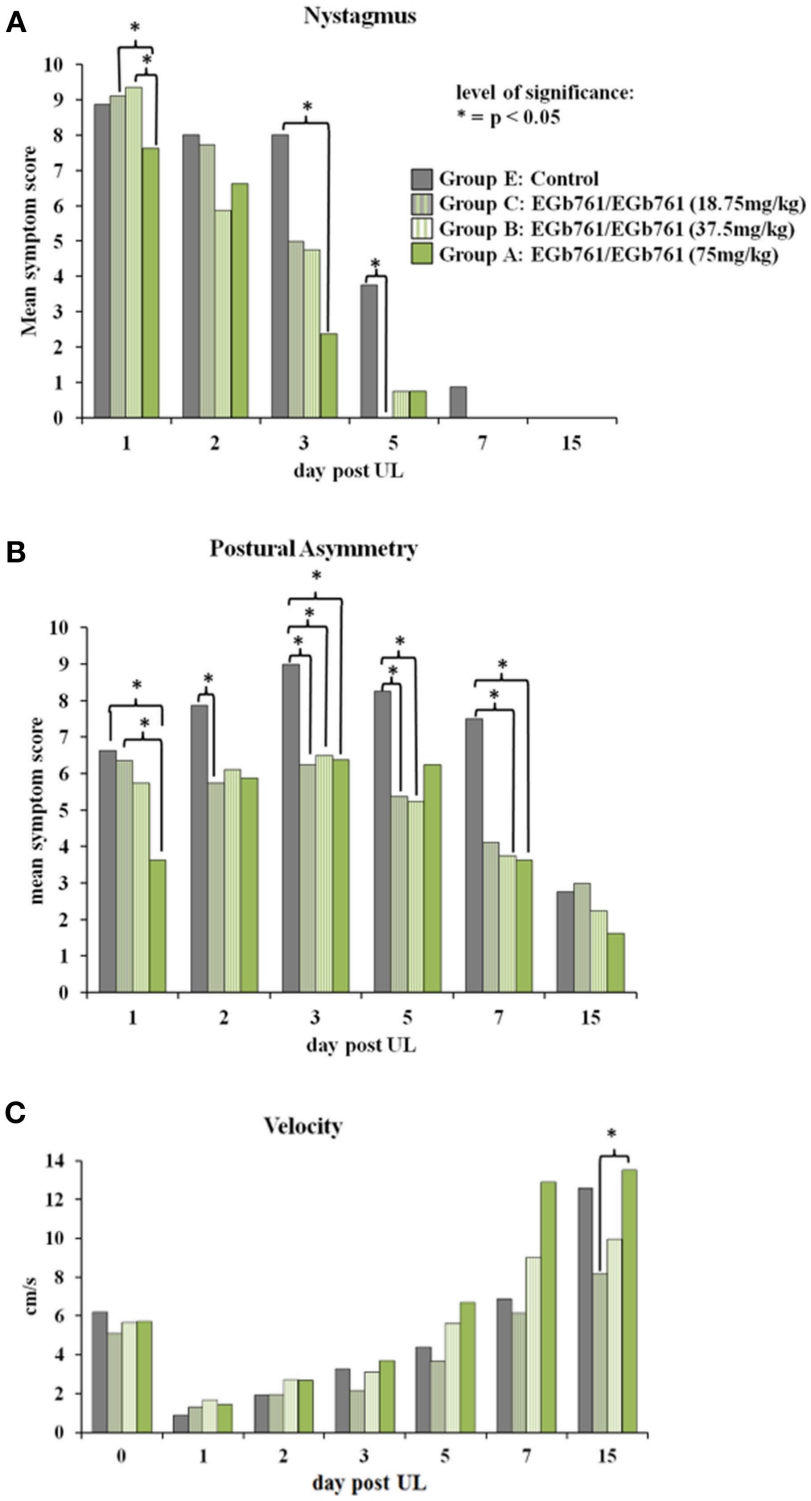
Dose-dependency of EGb 761 on behavioral scores of vestibular imbalance and locomotor velocity. **(A)** Nystagmus scores were significantly lower on day 1 in the EGb 761/EGb 761 75 mg/kg group A compared to groups B and C and on day 3 in the EGb 761 group A compared to Controls. The EGb 761/EGb 761 18.75 mg/kg group C had lower nystagmus scores than Controls on day 5 post UL. **(B)** Postural asymmetry scores were lower in the EGb 761/EGb 761 75 mg/kg group A on days 1, 3, and 7, in the EGb 761/EGb 761 37.5 mg/kg group B on days 3, 5 and 7 and in the EGb 761/EGb 761 18.75 mg/kg group C on days 2, 3, and 5 post UL relative to Controls. Group A had lower nystagmus scores on day 1 post UL compared to group C. **(C)** Open field testing indicated a significant increase in locomotor velocity on day 15 post UL in the EGb 761/EGb 761 75 mg/kg group A compared to the 18.75 mg/kg group C. Asterisk indicates a level of significance in Kruskal-Wallis *post hoc* test of *p* < 0.05.

Dose-response experiments in groups treated with EGb 761 doses of 75 mg/kg (group A), 37.5 mg/kg (group B), 18.75 mg/kg (group C), or Control food (group E) 6 weeks prior and 15 days post UL showed the following results: For nystagmus scores Kruskal-Wallis H test indicated significant differences between groups on day 1 (χ^2^ = 13.3, *p* = 0.01), day 3 (χ^2^ = 14.2, *p* = 0.007), and day 5 (χ^2^ = 9.8, *p* = 0.043). Nystagmus scores were significantly lower in the EGb 761/EGb 761 75 mg/kg (group A) vs. 37.5 mg/kg (group B) (*z* = 19.4, *p* = 0.01), as well as 18.75 mg/kg (group C) (*z* = 16.6, *p* = 0.047) on day 1 only, whereas there were no significant differences between the treatment groups A, B and C at later time points (Figure 4A). Postural asymmetry scores were significantly different between groups on day 1 (χ^2^ = 16.9, *p* = 0.002), day 2 (χ^2^ = 11.1, *p* = 0.025), day 3 (χ^2^ = 15.4, *p* = 0.004), day 5 (χ^2^ = 12.2, *p* = 0.016), and day 7 (χ^2^ = 12.9, *p* = 0.012). Postural asymmetry scores were lower in the EGb 761/EGb 761 75 mg/kg (group A) vs. the 18.75 mg/kg (group C) (z = 19.6, *p* = 0.011) and the Control group E (z = 22.5, *p* = 0.002) on day 1. While the postural asymmetry scores for treatment groups A, B and C vs. Control group E were lower on days 2, 3, 5, and 7, with some comparisons reaching significance (Figure 4B), again there were no significant differences between the EGb 761 groups at later time points. There were no significant group differences for head roll tilt or elevated tail rotation scores on any time point. Open field testing indicated a significant difference in velocity (χ^2^ = 9.9, *p* = 0.042) and moving duration between group (χ^2^ = 10.8, *p* = 0.03) only on day 15 post UL. *Post hoc* testing revealed that the EGb 761/ EGb 761 high dose group A had significantly increased locomotor velocity (*z* = 17.6, *p* = 0.04) and moving duration (*z* = 19.1, *p* = 0.018) compared to the low dose group C (Figure 4C). Duration of stay in the open field central zone was significantly different between groups on day 5 (χ^2^ = 15.1, *p* = 0.005) and day 7 post UL (χ^2^ = 16.2, *p* = 0.002). In *post hoc* analysis on day 5 (*z* = 21.2, *p* = 0.005) and day 7 (*z* = 21.1, *p* = 0.002) the EGb 761/EGb 761 75 mg/kg (group A) stayed longer in the central zone compared to the 18.75 mg/kg (group C) (data not shown).

### Blood Levels of Bilobalide, Ginkgolides A and B Correlate With Behavioral Parameters

**Figure 5:**
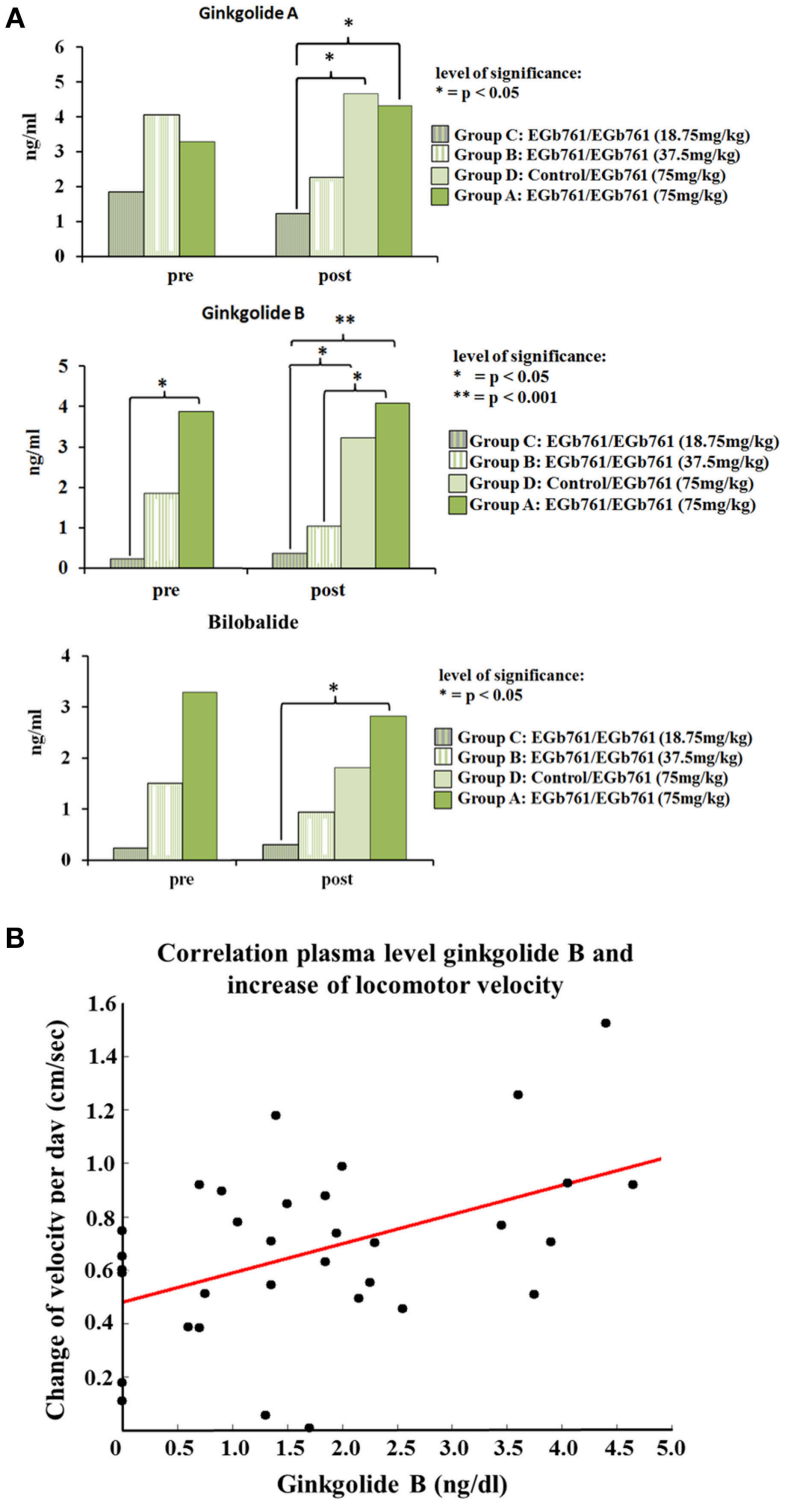
Plasma levels of EGb 761 substrates and correlation to behavioral scores. **(A)** Before UL, plasma levels of ginkgolide A and bilobalide were significantly higher in the EGb 761/EGb 761 group A and B compared to Controls. Ginkgolide B levels were higher in the group A compared to group C. Fifteen days post UL, ginkgolide A and B levels were significantly higher in the EGb 761/EGb 761 75 mg/kg group A and the Control/EGb 761 group D compared to the 18.75 mg/kg group C. Additionally, gingkolide B levels were higher in the EGb 761/EGb 761 75 mg/kg group A compared to the 37.5 mg/kg group B. Levels of bilobalide were significantly higher in the EGb 761/EGb 761 75 mg/kg group A compared to the group C. **(B)** Locomotor velocity after UL moderately but significantly increased over time with higher plasma levels of ginkgolide B (R = 0.46, *p* = 0.0068). Asterisk indicates a level of significance in Kruskal-Wallis *post hoc* test of *p* < 0.05.

Plasma levels of the terpene lactones bilobalide and ginkgolides A and B were as follows: prior to UL, Kruskal-Wallis H test revealed a significant difference for bilobalide (χ^2^ = 38.0, *p* < 0.001), ginkgolide A (χ^2^ = 38.7, *p* < 0.001), and ginkgolide B plasma levels (χ^2^ = 30.6, *p* < 0.001). *Post hoc* analysis showed significantly higher plasma levels for bilobalide and ginkgolide A in the EGb 761/EGb 761 75 mg/kg (group A) and 37.5 mg/kg (group B) compared to the Control group E and the Control/ EGb 761 group D, respectively. Ginkgolide B levels additionally were higher in the EGb 761/EGb 761 groups A compared to group C. On day 15 post UL, bilobalide (χ^2^ = 34.7, *p* < 0.001), ginkgolide A (χ^2^ = 35.7, *p* < 0.001), and ginkgolide B (χ^2^ = 30.6, *p* < 0.001) were significantly different between groups. Bilobalide levels were higher for the EGb 761/EGb 761 group A (*z* = 31.9, *p* < 0.001) and B (*z* = 17.1, *p* = 0.04), but not C, and the Control/EGb 761 group D vs. the Control group E (*z* = 24.4, *p* < 0.001). Additionally, higher bilobalide levels were found in EGb 761/EGb 761 group A vs. group C (*z* = 22.9, *p* = 0.002). For ginkgolide A, plasma levels in the EGb 761/EGb 761 group A (*z* = 27.1, *p* < 0.001) and Control/EGb 761 group D (*z* = 29.1, *p* < 0.001) were significantly higher than in Controls and the EGb 761/EGb 761 group C (*z* = 18.5, *p* = 0.027; *z* = 20.4, *p* = 0.003). Finally, ginkgolide B plasma levels were higher in the EGb 761/EGb 761 group A (*z* = 27.6, *p* < 0.001) and Control/EGb 761 group D (*z* = 21.2, *p* = 0.001) vs. the Control group E, as well as relative to EGb 761/EGb 761 group C (*z* = 24.5, *p* < 0.001; *z* = 18.1, *p* = 0.009). Levels in group A were significantly higher than in group B (*z* = 20.0, *p* = 0.008) (Figure 5A).

Correlations of plasma levels for bilobalide, ginkgolide A and B with the slope of locomotor velocity, nystagmus, or postural asymmetry over time revealed the following results: Locomotor velocity after UL moderately, but significantly increased over time with higher plasma levels of ginkgolide A (*R* = 0.47, *p* = 0.0062), ginkgolide B (*R* = 0.46, *p* = 0.0068), and bilobalide (*R* = 0.35, *p* = 0.047) (Figure 5B). The slope of postural compensation over time showed some mild but not significant correlation with plasma levels of ginkgolide A and B (*R* = −0.25) as well as bilobalide (*R* = −0.30). Nystagmus compensation did not correlate to plasma levels of terpene lactones.

### Dose-Dependent Effects of EGb 761 on Regional Cerebral Glucose Metabolism After UL

**Figure 6:**
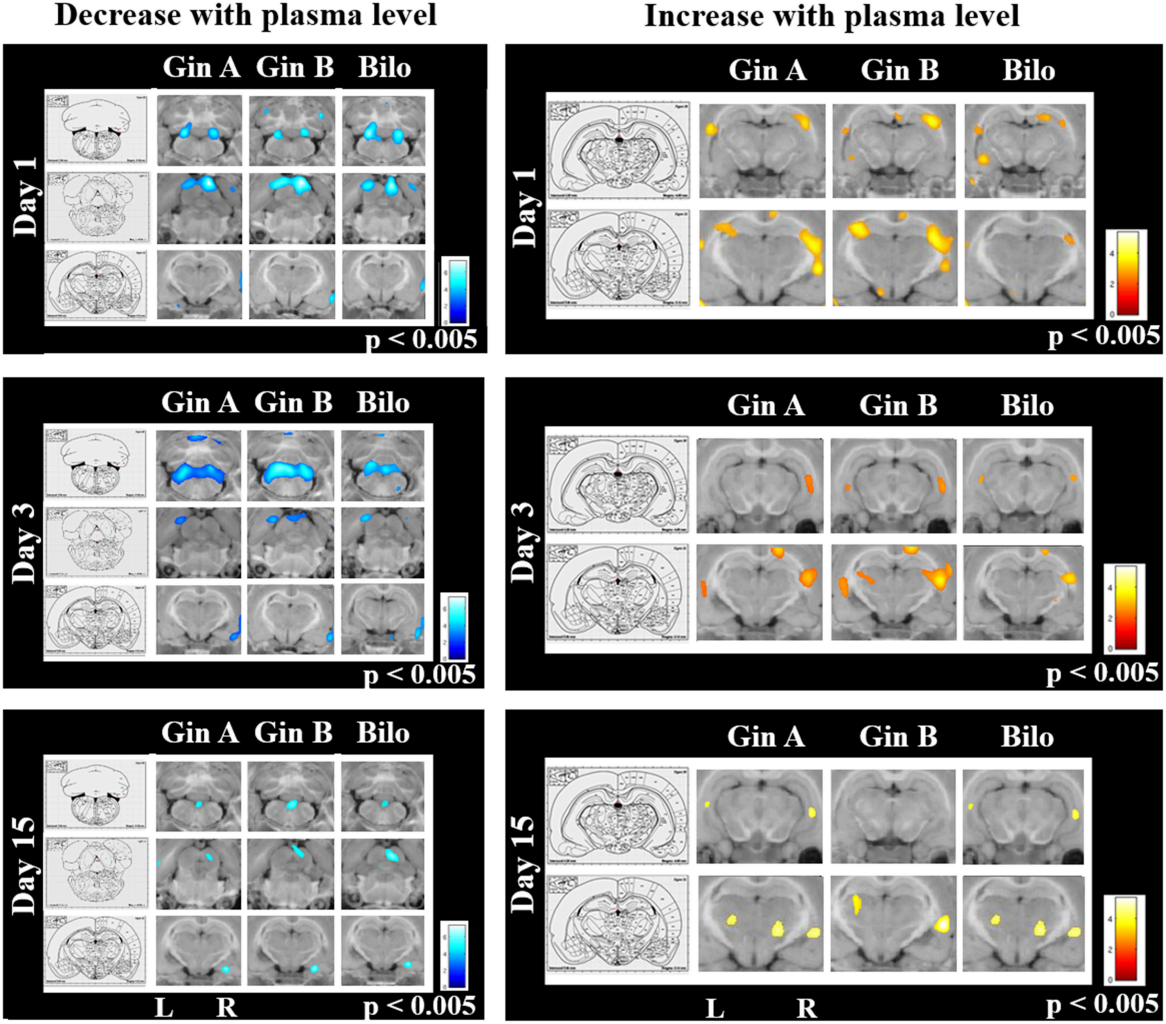
Correlation analysis of regional cerebral glucose metabolism and plasma levels for ginkgolide A, ginkgolide B, and bilobalide on days 1, 3, and 15 post UL. On day 1, rCGM decreased with higher ginkgolide A, ginkgolide B, and bilobalide levels, respectively, in the vestibular nuclei, cerebellum and inferior colliculi on both sides and right temporoparietal cortex (upper row, left). An rCGM increase with plasma levels was found in the hippocampus (lateral C2/3 region bilaterally) (upper row, right). On day 3, a decrease of rCGM appeared in the vestibular nuclei, the cerebellum and right temporoparietal cortex and an increase in the hippocampus bilaterally with higher plasma levels of ginkgolides and bilobalide (middle row). On day 15, rCGM relatively decreased with increasing plasma levels of ginkgolide A, B, and bilobalide between the vestibular nuclei (commissural system), in the right inferior colliculus and right amydala (lower row, left). rCGM increased in the lateral hippocampus and thalamus with higher levels of ginkgolide A, ginkgolide B, and bilobalide on this day (lower row, right). Level of significance: *p* < 0.005. L, left, R, right; Gin A, ginkgolide A; Gin B, ginkgolide B; Bilo, bilobalide.

Correlation analysis of increases and decreases of regional cerebral glucose metabolism (rCGM) to the individual plasma levels of ginkgolide A, B and bilobalide on days 1, 3, and 15 post UL in all animals of the EGb 761 75, 37.5, and 18.75 mg/kg groups A, B, and C revealed the following patterns: On day 1, rCGM decreased with higher ginkgolide A, ginkgolide B, and bilobalide levels, respectively, in the vestibular nuclei, cerebellum and inferior colliculi on both sides and right temporoparietal cortex. An rCGM increase with plasma levels was found in the hippocampus (lateral C2/3 region bilaterally). On day 3, a decrease of rCGM appeared in the vestibular nuclei, the cerebellum and right temporoparietal cortex and an increase in the hippocampus bilaterally with higher plasma levels of ginkgolides and bilobalide. On day 15, rCGM relatively decreased with increasing plasma levels of ginkgolide A, B and bilobalide between the vestibular nuclei (commissural system), in the right inferior colliculus and right amydala. rCGM increased in the lateral hippocampus and thalamus with higher levels of ginkgolide A, ginkgolide B and bilobalide on this day (*p* < 0.005, uncorrected) (Figure 6).

Comparison of the EGb 761/EGb 761 75, 37.5, and 18.75 mg/kg groups (groups A, B, and C) to the Control group (group E) revealed the following rCGM decreases and increases (*p* < 0.005, uncorrected): In the EGb 761/EGb 761 75 mg/kg (group A), rCGM was significantly decreased in the cerebellum on day 1 (paraflocculus and lobulus simplex), day 3 (nodulus), and day 15 (right vermis) post UL compared to the Control group E. In the EGb 761/EGb 761 37.5 mg/kg (group B), rCGM was significantly reduced in the vestibular nuclei and cerebellum on day 1 (paraflocculus bilaterally), cerebellum on day 7 (right paraflocculus), and day 15 post UL (right vermis) relative to Controls. In the EGb 761/EGb 761 18.75 mg/kg group C, rCGM was lower in the cerebellum (nodulus) on day 3 post UL compared to the Control group E (Figure S1, left side).

In the EGb 761/EGb 761 75 mg/kg (group A) a significant increase of rCGM appeared in the hippocampus (lateral CA2/3 region bilateral and fibria of the hippocampus right) on day 1 post UL, which decreased until day 3 (lateral CA2/3 region and fimbria of the hippocampus right) (compared to the Control group E, respectively). In the EGb 761/EGb 761 37.5 mg/kg (group B) there was no hippocampal activation over time compared to Controls. In the EGb 761/EGb 761 18.75 mg/kg (group C) a significant rCGM increase was only found in the left posterolateral thalamus on day 1 post UL compared to Controls. On days 3 and 7 post UL, there were no significant rCGM increases in this group. On day 15 post UL rCGM was increased in the right amygdaloid nucleus in the low-dose group (group C) compared to Controls (Figure S1, right side).

## Discussion

The major findings of this study were the following: (1) Oral application of *Ginkgo biloba* extract EGb 761 accelerates compensation of nystagmus and postural asymmetry and improves mobility after unilateral labyrinthectomy in the rat. (2) Plasma levels of ginkgolide A, ginkgolide B, and bilobalide correlate significantly with an increase in spontaneous locomotor velocity over time. (3) EGb 761 improves vestibular compensation also when started after onset of vestibular imbalance. (4) EGb 761 modulates cerebral glucose metabolism in the vestibular nuclei, cerebellar, thalamic, hippocampal, and temporoparietal networks dose-dependently after unilateral labyrinthectomy.

### Differential and Dose-Dependent Effects of EGb 761 Extract on Behavioral Vestibular Compensation

The polyvalent *Ginkgo biloba* extract EGb 761 has been in clinical use for the treatment of vestibular disorders for decades (26–28). Previous animal experiments have reported improvement of symptoms after unilateral vestibular damage by EGb 761: In the rat model of chemical UL, EGb 761 application (50 mg/kg i.p. for 10 weeks) improved recovery of nystagmus and static postural symptoms (16). Similarly, Lacour et al. showed an acceleration of postural and locomotor balance recovery as well as earlier restoration of vestibulo-collic reflex after UL in the cat by intraperitoneal injection of 50 mg/kg EGb 761 over 30 days (17). In the guinea pig UL model, the EGb 761 component ginkgolide B improved compensation of spontaneous nystagmus at a dose of 25 mg/kg i.p., but not at higher doses (18). Schlatter et al. criticized these studies for the lack of either a control group with solution vehicle only or dose-response groups (29). In their experiments, i.p. application of EGb 761 at doses of 25, 50, and 100 mg/kg for 3 days after UL in the guinea pig failed to induce significant dose-dependent improvements of static vestibular symptoms as compared to vehicle application (29). The discrepancy of findings was attributed partly to differences in time course of application and effects of the vehicle. Furthermore, it was speculated that EGb 761 may have differential effects on compensation of static and dynamic vestibular imbalance. To address these open questions, we conducted a study in the rat model, which used an alternative mode of application via the oral route, examined compensation of both static and dynamic signs of vestibular asymmetry, compared effects of dose and time course of EGb 761 administration, controlled plasma levels of the most important EGb 761 substrates, and correlated them to behavior and cerebral glucose metabolism. In our study, oral administration of EGb 761 improved compensation of static signs of vestibular asymmetry, such as nystagmus (vestibulo-ocular motor function) and postural imbalance (vestibulo-spinal function), as well as of dynamic function indicated by an increase in spontaneous mobility and locomotor velocity (Figures 2, 3). The effects of EGb 761 on static compensation appeared earlier than the effects on dynamic compensation. The beneficial effects on recovery of nystagmus and postural asymmetry persisted, when EGb 761 administration started at the time of UL, suggesting immediate alongside more delayed effects of the extract on vestibular compensation (Figure 2). Unspecific effects of a solution vehicle (as suggested for DMSO) or byproducts were unlikely, because EGb 761 was applied orally, and treatment and control groups differed in their time course of compensation (18). Effects of EGb 761 on vestibular compensation were partially dose-dependent in our study: decrease of nystagmus and postural asymmetry on day 1 post UL was significantly higher for the 75 mg/kg than the 18.75 mg/kg group. Locomotor velocity and mobility improved more in the high-dose EGb 761 group compared to the low-dose group on day 15 post UL. In contrast, postural asymmetry on day 3 post UL decreased to an equal extent in all EGb 761 groups and on day 7 post UL in the EGb 761 high- and middle-dose group, suggesting some saturation of the dose-response effect for this parameter.

### EGb 761 Mechanisms of Action on Vestibular Compensation: Chemical Substrates and Cerebral Networks

The effects of EGb 761 on various mechanisms of neuroplasticity, including changes in cellular excitability, synaptic function, neurito- and neurogenesis, were reported previously (20, 30, 31). The specific mode of action of EGb 761 on central plasticity following vestibular lesions remains to be determined. The most important open questions are: which substrates of the polyvalent EGb 761 extract modulate vestibular compensation at which dose, which cerebral networks are involved, and which mechanisms of central plasticity (early vs. delayed) are affected by EGb 761. The EGb 761 extract contains two well-characterized biochemical components (terpene lactones and flavonoids). Flavonoids are abundantly present in herbs and vegetables, whereas terpene lactones (e.g., bilobalide, ginkgolide A and B) are components specific to *Ginkgo biloba* (30, 32). The special extract of *Ginkgo biloba* leaves–EGb 761–contains standardized amounts of flavonoids (22–27%) and terpene lactones (5–7%). In a previous study, ginkgolide B but not A effectively reduced nystagmus after UL in the guinea pig (18). Compensation of postural asymmetry or head roll tilt was not affected by either substrate. Another study suggested that the non-terpenic fraction of the polyvalent EGb 761 extract was pharmacologically active in compensation of locomotor dysequilibrium after UL in cats (33). However, non-specific effects of the solution vehicle could not be excluded (29). The correlation analysis of terpene lactone plasma levels and behavior in the current study showed differential effects of EGb 761 substrates: higher ginkgolide A and B and, to a lesser extent, bilobalide plasma levels significantly correlated with a faster increase in locomotor velocity over time after UL. Plasma levels of bilobalide more than gingkolide A and B tended to inversely correlate with the slope of postural compensation. In contrast to data reported by Maclennan and colleagues, no significant correlations were found between compensation of nystagmus and terpene lactones plasma levels. Based on our data, one can assume that EGb 761 components have pleiotrophic effects on static and dynamic vestibular compensation.

The questions remain, in which cerebral networks and by which mechanisms EGb 761 improves vestibular compensation after inner ear lesions. Overall, CNS uptake of EGb compounds ginkgolide A, B, and bilobalide is rapid after oral administration in the rat, whereas ginkgolide C does not penetrate the blood-brain barrier (15). About 60% of radiolabeled EGb 761 passes into the brain within 72 h after oral application (34). About 90% of EGb 761 substrates are distributed in the hippocampus, frontal cortex, striatum, and cerebellum (35). Previous experiments on the modes of action of EGb 761 in vestibular animal models mostly have focused on the vestibular nuclei. Yabe et al. showed a suppression of the horizontal vestibulo-ocular reflex gain in normal guinea pigs 2 h after i.p. injection of EGb 761 at a dose of 50 mg/kg (36). Ginkgolide B (25 mg/kg i.p. single dose) accelerated suppression of spontaneous nystagmus in a UL guinea pig model, suggesting action on the level of the vestibular nuclei (37). Functionally, it was hypothesized that EGb 761 may modulate neuronal excitability in the vestibular nuclei (36). Lacour et al. identified structural changes at the level of the vestibular nuclei induced by EGb 761 in UL cats, which consisted of a more rapid synaptic reoccupation in the deafferented medial vestibular nucleus (17). Another study reported an increase of protein synthesis on a brainstem level by EGb 761 (38). The time constant of the latter effects however exceeded the behavioral compensation of vestibular imbalance (17). These data therefore suggest that EGb 761 extract has immediate functional effects on vestibular asymmetry and delayed structural effects on consolidation of vestibular networks. In the current study, regional cerebral glucose metabolism decreased in the vestibular nuclei and cerebellum with higher plasma levels of ginkgolides and bilobalide on days 1 and 3 post UL, following the temporal dynamics of compensation of static vestibular functions. In accordance, a previous autoradiographic study in the rat also revealed a significant decrease of glucose consumption in the cerebellar cortex and pons following application of EGb 761 (50 mg/kg dose p.o. for 15 days) (39). One can only speculate whether the decreased glucose uptake in the vestibular nuclei reflects a regional reduction of neuronal activity or excitability by changes in membrane properties or neurotransmission. From a functional perspective, a reduced excitability in the vestibular nuclei on both sides would help to restore vestibular symmetry and thus would contribute to static vestibular compensation. The second significant effect of EGb 761 on cerebral glucose consumption was an increased uptake in the hippocampus in the high-dose group until day 7 post UL (Figure 6). The hippocampus is a well-known target for EGb 761 action and a hub of the cerebral vestibular networks (5, 20, 40). EGb 761-induced changes of hippocampal long-term potentiation, neurito- and neurogenesis are documented (20). The time course of glucose changes in the UL model would point toward a change in hippocampal neuronal activity rather than structural changes on network levels. Accordingly, spatial exploration behavior in the open field significantly differed in the EGb 761 (75 mg/kg) group and controls on days 3, 5, and 7 post UL. A longer duration of in-center stays would indicate reduced spatial anxiety and increased interest in spatial exploration. One cannot exclude the possibility that the altered hippocampal glucose consumption in the high-dose EGb 761 group was rather a consequence of improved overall mobility.

### Translational Perspectives for the Use of EGb 761 in Vestibular Disorders

In pharmacologic research the translation of animal data into the clinical context can be limited by differences in the route, time course and dose of application, metabolism and neurobiology between species. In the current study, we chose oral EGb 761 administration and included a starting point for treatment after onset of vestibular imbalance to resemble the clinical situation of a patient with an acute unilateral vestibulopathy. Effective plasma levels for bilobalide, ginkgolide A and B in our high-dose EGb 761 group (75 mg/kg) were similar to previously reported values in healthy humans taking a maximal standard dose of approved *Ginkgo biloba* extract drugs (25). Indeed, controlled clinical studies have also reported benefits of EGb 761 on vestibular compensation: Haguenauer et al. described a significant improvement of subjective vertigo symptoms and quality of life in 70 patients with unilateral vestibulopathies after 3 months of EGb 761 treatment as compared to placebo (28). Other clinical trials showed an augmentation of exercise-induced postural recovery by EGb 761 vs. placebo administration in patients with vestibular disorders (41, 42). It therefore seems likely that the experimental effects of EGb 761 in vestibular animal models can, in principle, be translated to patients with vestibular disorders (19). Further controlled studies will have to be conducted in order to determine the optimal time course and dose of EGb 761 administration in patients with acute and chronic vestibulopathies.

EGb 761 induced changes of regional cerebral glucose metabolism in the vestibular nuclei, vestibulocerebellum, thalamus, hippocampus, and temporoparietal cortex after UL in the rat model. From a translational perspective, EGb 761 seems to target vestibular hubs, which are also found to be modulated in patients with acute unilateral vestibular syndromes: in acute vestibular neuritis, an increase of glucose metabolism was reported in the pontomesencephalic brainstem, posterolateral thalamus, parietoinsular vestibular cortex and hippocampus (43). In patients with Wallenberg syndrome, vestibulocerebellar activations were found during the acute stage (44). These regions show a striking overlap with the networks and hubs modulated by EGb 761 in the rat model of vestibular imbalance.

### Limitations of the Study

The relatively short follow-up post UL may not be sufficient to pick up delayed effects of EGb 761 administration on vestibular compensation and plasticity. As the method of serial [^18^F]-FDG-μPET imaging is concerned, the authors are aware that changes in glucose metabolism do not allow conclusions on molecular mechanisms underlying EGb 761 effects. Correlations of terpene lactone plasma levels with behavioral parameters of vestibular compensation may not be misinterpreted as a proof of causation. Plasma levels of flavonoids were not measured. A strict dose-dependency could not be proven for all read-out parameters tested. It was present for nystagmus and postural asymmetry only on day 1 post UL and for locomotor velocity on day 15 post UL. For the intermediate time points dose-response effects were less clear. Correlation of plasma levels of ginkgolides and bilobalide with cerebral glucose consumption, however, showed a clear dose-dependency for effects in the vestibular nuclei, cerebellum and hippocampus on day 1, 3, and 15 post UL.

### Conclusions

The current study shows beneficial effects of the oral *Ginkgo biloba* extract EGb 761 application on compensation of static and dynamic vestibular function following inner ear damage. Improvement of nystagmus, postural asymmetry, and locomotor behavior indicates a modulation of vestibulo-ocular motor, vestibulo-spinal, and cortico-striatal networks. Changes in spatial exploration behavior suggest additional EGb 761 effects on vestibulo-hippocampal circuits. Terpene lactones like ginkgolide A and B as well as bilobalide probably account for some of the effects on mobility, whereas compensation of nystagmus and postural asymmetry may involve other pharmacologically active substrates of the polyvalent extract. This rat study supports the translational approach of using EGb 761 at higher dosages for medical acceleration of vestibular compensation in surgical or disease related acute vestibular loss. Serial imaging of cerebral glucose metabolism indicates a modulation of the vestibular nuclei, cerebellar, thalamic, hippocampal and temporoparietal networks by EGb 761 during vestibular compensation. The observed mechanisms might account for some of the EGb 761 benefits reported in controlled clinical trials in patients with vestibular disorders.

## Author Contributions

ML: acquisition of data, analysis and interpretation of data, statistical analysis; AG: analysis and interpretation of data, statistical analysis; EE, CB, and AK: acquisition of data, analysis and interpretation of data; RO: acquisition of data; EW, SZ, and PB: study concept and design, revising the manuscript; TB: interpretation of data, revising the manuscript; MD: study concept and design, analysis, and interpretation of data, revising the manuscript; AZ: drafting, revising the manuscript, study concept and design, acquisition of data, analysis and interpretation of data, statistical analysis.

### Conflict of Interest Statement

AZ received speaker's honoria from Dr. Willmar Schwabe GmbH. The remaining authors declare that the research was conducted in the absence of any commercial or financial relationships that could be construed as a potential conflict of interest.

## Footnotes

^1^EGb 761® is the active ingredient of Tebonin® intens, Dr. Willmar Schwabe Pharmaceuticals, Karlsruhe, Germany.

## Supplementary Material

Figure S1 Sequential increases and decreases of the cerebral glucose metabolism in the EGb 761 treated groups relative to controls. In the EGb 761/EGb 761 75 mg/kg group rCGM was significantly decreased in the cerebellum on days 1, 3 and day 15 post UL as well as in the ventral intermediate thalamus on day 1 post UL compared to the Control group. In the EGb 761/EGb 761 37.5 mg/kg group B, rCGM was significantly reduced in the vestibular nuclei and cerebellum on day 1, 7, and 15 as well as in the ventrointermediate thalamus on days 1 and 3 post UL as compared to Controls. In the EGb 761/EGb 761 18.75 mg/kg group, rCGM was lower in the cerebellum on day 3 post UL compared to the Control group E. In the EGb 761/EGb 761 75 mg/kg group a significant increase of rCGM appeared in the hippocampus on day 1 post UL, which decreased until day 3 compared to the Control group, respectively. In the EGb 761/EGb 761 37.5 mg/kg group there was no hippocampal activation over time, but instead an rCGM increase in the left striatum on day 1, left thalamus and right striatum on day 3 post UL (compared to Controls). In the EGb 761/EGb 761 18.75 mg/kg group C a significant rCGM increase was only found in the left posterolateral thalamus on day 1 post UL compared to Controls. On day 15 post UL rCGM was increased in the right amygdaloid nucleus in the low-dose group compared to Controls. Level of significance: *p* < 0.005. L, left; R, right.
