# Supplementary figures and images for: Ginkgo biloba Extract EGb 761 Improves Vestibular Compensation and Modulates Cerebral Vestibular Networks in the Rat
Source: Front Neurol. 2019 Feb 25;10:147. doi: 10.3389/fneur.2019.00147 (PMC6397839; doi:10.3389/fneur.2019.00147)

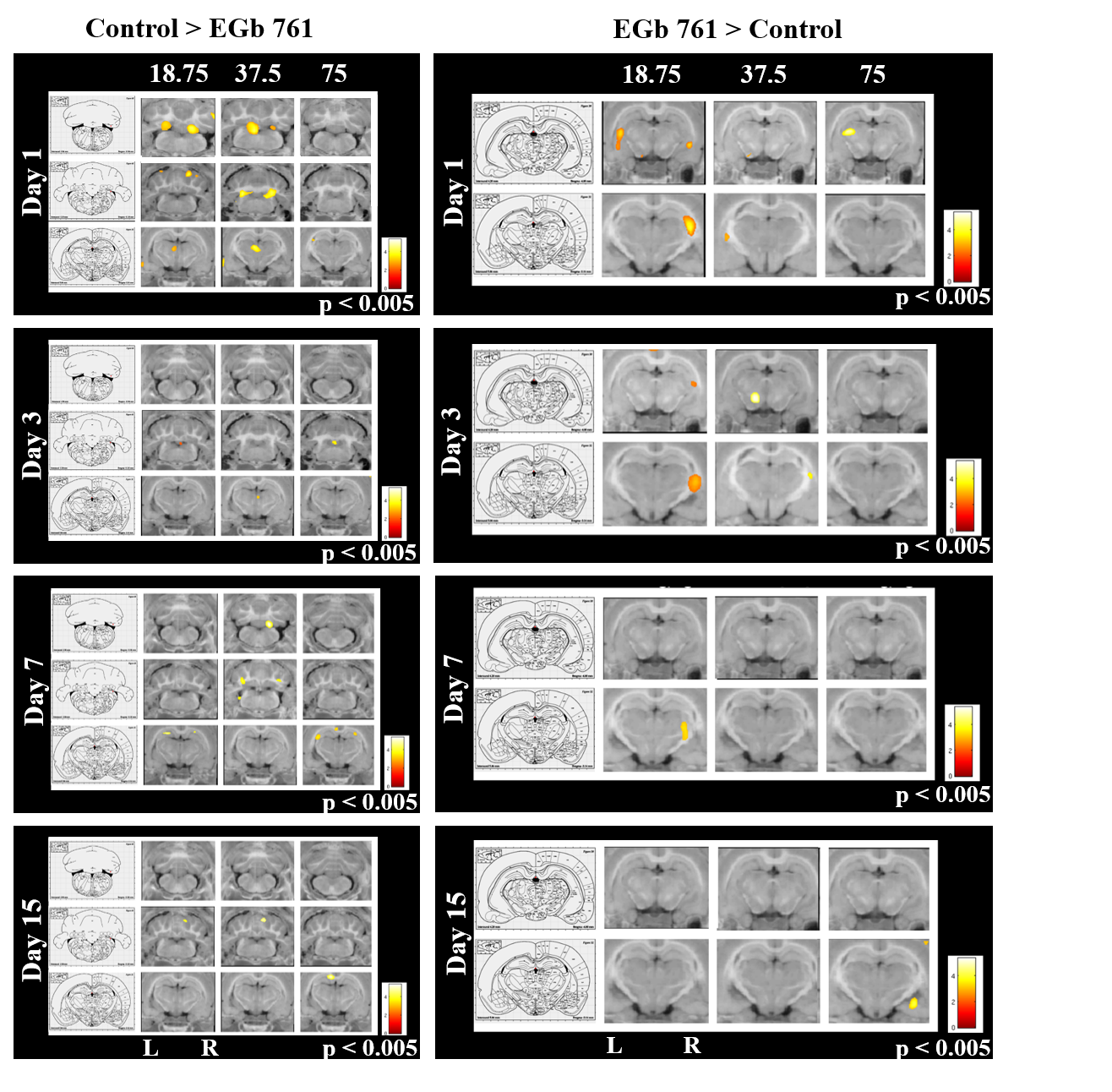

Supplement: Figure S1 — Sequential increases and decreases of the cerebral glucose metabolism in the EGb 761 treated groups relative to controls. In the EGb 761/EGb 761 75 mg/kg group rCGM was significantly decreased in the cerebellum on days 1, 3 and day 15 post UL as well as in the ventral intermediate thalamus on day 1 post UL compared to the Control group. In the EGb 761/EGb 761 37.5 mg/kg group B, rCGM was significantly reduced in the vestibular nuclei and cerebellum on day 1, 7, and 15 as well as in the ventrointermediate thalamus on days 1 and 3 post UL as compared to Controls. In the EGb 761/EGb 761 18.75 mg/kg group, rCGM was lower in the cerebellum on day 3 post UL compared to the Control group E. In the EGb 761/EGb 761 75 mg/kg group a significant increase of rCGM appeared in the hippocampus on day 1 post UL, which decreased until day 3 compared to the Control group, respectively. In the EGb 761/EGb 761 37.5 mg/kg group there was no hippocampal activation over time, but instead an rCGM increase in the left striatum on day 1, left thalamus and right striatum on day 3 post UL (compared to Controls). In the EGb 761/EGb 761 18.75 mg/kg group C a significant rCGM increase was only found in the left posterolateral thalamus on day 1 post UL compared to Controls. On day 15 post UL rCGM was increased in the right amygdaloid nucleus in the low-dose group compared to Controls. Level of significance: p < 0.005. L, left; R, right. [file Image_1.TIF]
